# Prioritising the development of severity distributions in burden of disease studies for countries in the European region

**DOI:** 10.1186/s13690-019-0385-6

**Published:** 2020-01-09

**Authors:** Grant M. A. Wyper, Ian Grant, Eilidh Fletcher, Neil Chalmers, Gerry McCartney, Diane L. Stockton

**Affiliations:** 1 Public Health Science Directorate, NHS Health Scotland, Meridian Court, 5 Cadogan Street, Glasgow, Scotland G2 6QE; 2 Information Services Division, NHS National Services Scotland, Gyle Square, 1 South Gyle Crescent, Edinburgh, Scotland EH12 9EB

**Keywords:** Severity distribution, Burden of disease, DALY, YLD, Summary measures of population health, SBOD, GBD, EBODN

## Abstract

Severity distributions are a means of summarising the range of health loss suffered to disease which enables estimates of disease occurrence to be paired with disability weights to estimate Years Lost to Disability (YLD) in burden of disease studies. There is a lack of current data exploring severity distributions, which has led to the Global Burden of Disease (GBD) study relying on using the same severity distributions across countries and regions across the world. This is also largely true for some national studies, although there are exceptions. Recent evidence has raised concerns that severity distributions are unlikely to be generalisable as major differences arise when using country-specific data to develop severity distributions. These issues raise uncertainties over interpreting YLD estimates, particularly if they are being used to develop and influence policies and to determine priorities across diseases and populations. It is clear that GBD researchers and those carrying out national studies need to work towards ensuring that estimates are based upon country-specific data, and, if possible, that the impact of assumptions are fully tested and understood. There is a lack of strategy about if, where, and how, this could be achieved, particularly around how efforts should be prioritised. This commentary advocates and presents a possible strategic approach to better understanding how efforts may be best placed.

## Background

Disability and functional loss that results from disease manifests in different people in different ways. In the case of a stroke survivor this can vary from being wheelchair-bound with cognitive loss and dependent on assistance to eat, dress or go to the toilet; to a stroke survivor that suffers no adverse long-term effects. Severity distributions are the means of summarising the range of health loss to disease to enable estimates of disease occurrence to be matched with relevant disability weights to estimate Years Lost to Disability (YLD). This is usually expressed as the proportion of cases living with either: mild, moderate, severe, or no health loss.

The Global Burden of Disease (GBD) study applies the same severity distributions to all countries and regions across the world, which are largely based on data from the United States and Australia [1]. This is due to a lack of data that can be used to estimate the distribution of severity in the occurrence of disease. The impact of this assumption has only recently been measured. Research from the Scottish Burden of Disease study illustrated that using the same overall estimate for the occurrence of disease can lead to large differences if the severity distribution is substantially different [2]. These results highlight that users of both GBD and national studies should be concerned about the applicability of these standard severity distributions across populations. It is not unreasonable to suggest that the effect may be amplified in low and middle income countries where the disability experienced from disease occurrence may be markedly different due to differences in disease severity, the effectiveness and accessibility of treatment, and the extent of social and service support available.

These issues raise uncertainties over the interpretation of YLD estimates, particularly when estimates are being used to develop and influence policies and to determine priorities across diseases and populations. Where possible, it is important that we work towards ensuring that estimates are based upon country-specific or generalisable data.

## Heterogeneity of approaches

Users of GBD estimates are using an assumption of fixed severity distributions across populations. Researchers from independent national studies have been left with either: using the same approach as the GBD study; or developing their own country-specific severity distributions for all, or a subset of, causes. Pivotal examples of this are found in South Korea and Germany, where researchers have opted to develop country-specific severity distributions [3, 4]. In Scotland, we have been fortunate in developing severity distributions for cancers, epilepsy and cirrhosis, but have currently used GBD data to infer severity distributions for all other causes due to limited study resources.

**Fig. 1:**
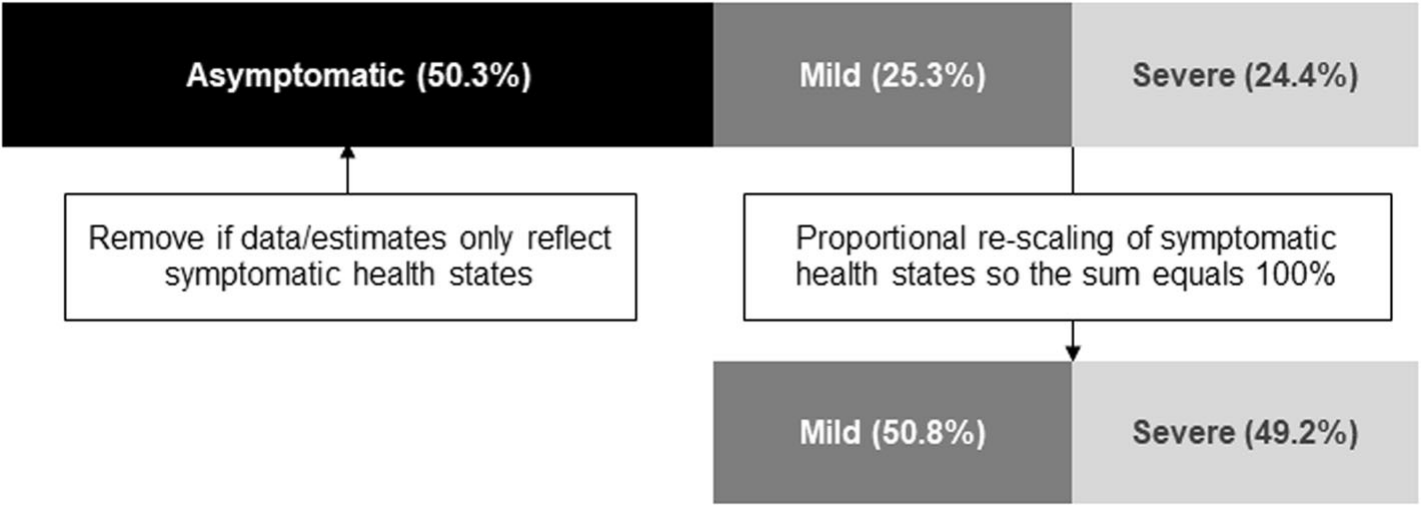
: Rescaling severity distributions that include asymptomatic cases to obtain symptomatic severity distributions

Practical solutions to refine GBD severity distributions can be achieved by working with data and clinical experts to generate proxy definitions for both symptomatic and asymptomatic health states. Asymptomatic health states are those that reflect a state of no health loss. The removal of asymptomatic health states allow for GBD severity distributions to be rescaled to focus solely on individuals that contribute to YLD estimates. We took this approach in Scotland for cocaine dependence (Fig. 1) as the context of the survey data which we used as an input was a good proxy for symptomatic cases. Although we could not further identify the moderate and severe health states with confidence, our estimate of symptomatic prevalent cases allowed us to remove our reliance on assumptions of the proportion of cases which were asymptomatic. This approach is only advised if the data captured is a robust and consistent proxy for health states representative of individual’s suffering from disease symptoms, and enables researchers to use the GBD severity distributions more flexibly.

Example derived using GBD 2016 global health state prevalence estimates for cocaine dependence.

## Establishing how efforts can be focused

A lack of evidence on impact of the choice of severity distributions across causes and populations suggests that current approaches remain speculative and efforts to test or improve upon them may represent an ineffective use of study resources. For users of GBD estimates, it is important that potential uncertainties around severity distributions are clear. Although uncertainty intervals are provided, and are helpful warnings for unstable estimates, they fail to capture other important sources of uncertainty inherent in estimates such as potential differences in culture, social values and healthcare access and effectiveness, which limit generalisability across countries and regions. Those working on independent studies need to understand whether there is additional merit in developing country-specific severity distributions, and for which causes, particularly if study resources are scarce.

**Fig. 2:**
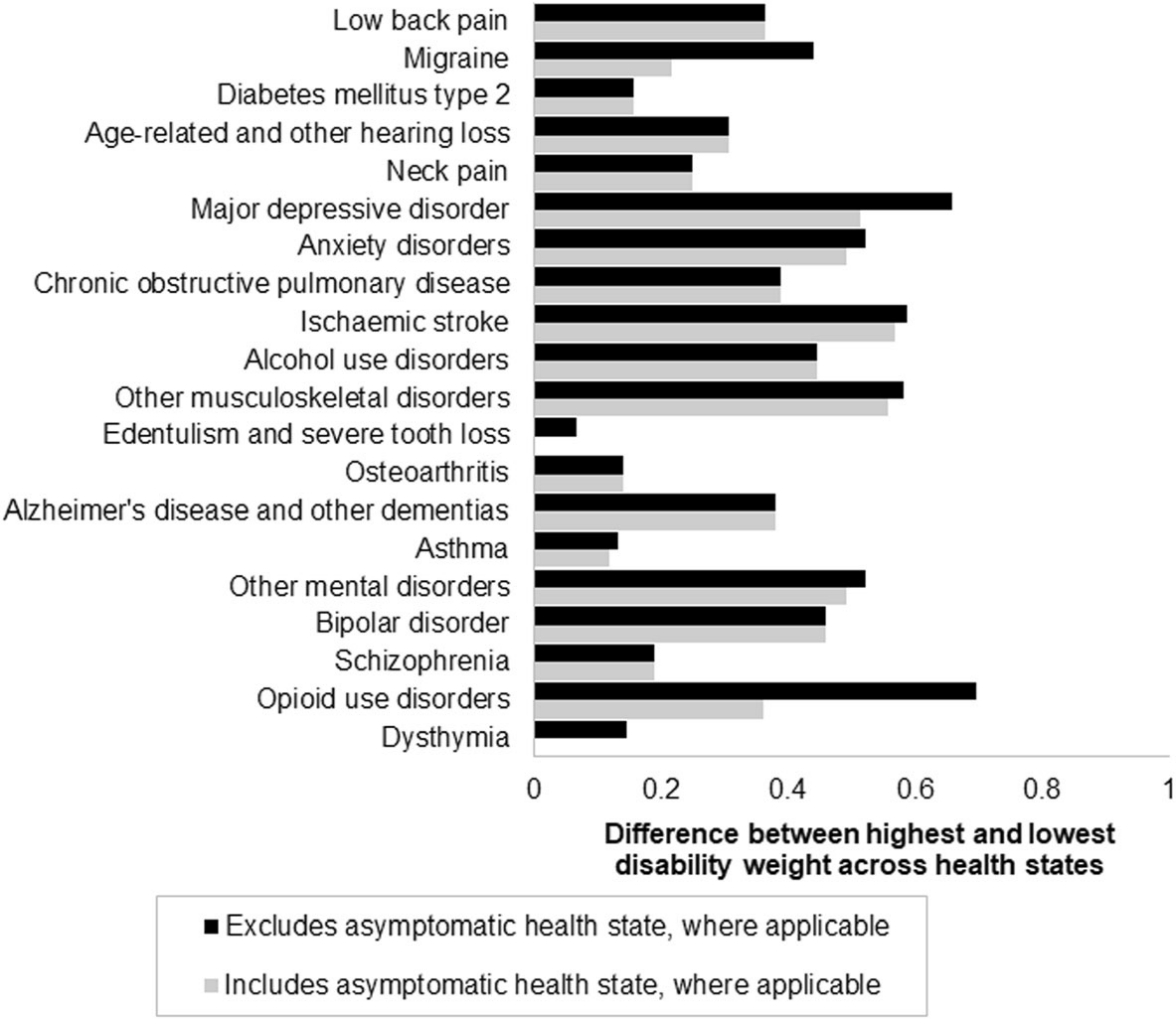
: Potential variation in disability weight for the 20 leading non-communicable diseases of YLD in the European region, 2017.

Collaborative efforts, experiences and techniques in developing and working with severity distributions from burden of disease researchers is a useful foundation for developing solutions. Structured environments which may give rise to these opportunities currently exist in forums such as the WHO Regional Office for Europe and Institute of Health Metrics and Evaluation’s jointly organised European Burden of Disease Network (EBODN), GBD Collaborator Portal or through the COST Action (CA18218) EU-European Burden of Disease Network [5–7]. Moreover, there are ways in which we can gain important inferences into where efforts should be targeted. A pragmatic start involves focusing on the 20 leading non-communicable diseases of YLD and their potential scale of variation in disability for the European region (Fig. 2). Health state disability weights from the GBD study allows us to understand which causes may result in the largest variation by taking the difference between the highest and lowest health state disability weight for a cause [8]. A potential limitation of this approach is that disability weight estimates, and other factors such as duration, are also subject to uncertainties such as differences in culture, social values and healthcare access and effectiveness.

YLD denotes Years Lost to Disability; 20 leading causes based on ranking of YLD rate for the European region; Non-communicable diseases only; Causes ordered ascendingly according to the number of YLD.

When covering all health states, including asymptomatic, the five diseases with the largest potential to be impacted by variation in disease severity are opioid use disorders, major depressive disorder, ischaemic stroke, other musculoskeletal disorders, anxiety and other mental disorders. If input data can be assumed to represent symptomatic populations only then the leading five priority diseases remain constant, with the exception of opioid use disorders, which has less potential to be impacted under this scenario.

## Conclusion

Assessing the leading causes of YLD and differences between the highest and lowest health state disability weights can be used to identify priority diseases for which it would be most beneficial to further develop severity distributions. Prioritising the development of severity distributions for conditions such as opioid use disorders, major depressive disorder, ischaemic stroke, other musculoskeletal disorders, anxiety and other mental disorders would help understand the wider uncertainties over applicability that are currently unanswered.

Practicalities over limited data availability often drive the direction of which work is undertaken. This approach considers the potential impact to focus on where research can add the most value. Although this may require additional time consuming fieldwork activities, or making changes to the way current data collection systems operate, it represents an opportunity to be more comfortable with the resulting YLD estimates that we are looking to use to influence policy-making.

Those working on national studies and GBD study collaborators should utilise forums such as EBODN, GBD Collaborator Portal and the EU-European Burden of Disease Network to work at scale to establish common strategies to developing disease coding phenotypes representative of health states to deliver higher quality generalisable definitions on disease severity.

## Data Availability

The datasets generated and/or analysed for this commentary are all publically available from: http://ihmeuw.org/4yy9, http://ghdx.healthdata.org/record/ihme-data/gbd-2017-disability-weights and http://ghdx.healthdata.org/record/ihme-data/gbd-2016-incidence-prevalence-and-ylds-1990-2016.

## Abbreviations

COST: European Cooperation in Science and Technology
EBODN: European Burden of Disease Network
EU: European Union
GBD: Global Burden of Disease
WHO: World Health Organization
YLL: Years Lost to Disability
