# Correction to: Prioritising the development of severity distributions in burden of disease studies for countries in the European region

**DOI:** 10.1186/s13690-020-00412-3

**Published:** 2020-04-02

**Authors:** Grant M. A. Wyper, Ian Grant, Eilidh Fletcher, Neil Chalmers, Gerry McCartney, Diane L. Stockton

**Affiliations:** 1 Public Health Science Directorate, NHS Health Scotland, Meridian Court, 5 Cadogan Street, Glasgow, Scotland G2 6QE; 2 Information Services Division, NHS National Services Scotland, Gyle Square, 1 South Gyle Crescent, Edinburgh, Scotland EH12 9EB

**Correction to: Arch Public Health (2020) 78: 3**

**https://doi.org/10.1186/s13690-019-0385-6**

In the original publication of this article [1], the author pointed out the legends of Figs. 1 and 2 are not entire. The correct legends are:

Fig. 1 Rescaling severity distributions that include asymptomatic cases to obtain symptomatic severity distributions. Example derived using GBD 2016 global health state prevalence estimates for cocaine dependence

Fig. 2 Potential variation in disability weight for the 20 leading non-communicable diseases of YLD in the European region, 2017. YLD denotes Years Lost to Disability; 20 leading causes based on ranking of YLD rate for the European region; Non-communicable diseases only; Causes ordered ascendingly according to the number of YLD

The publisher apologizes to the readers and authors for the inconvenience.
